# Sex and Ethnic Disparities in Stroke Revascularisation Treatments and Post-Stroke Outcomes in Patients with Heart Failure: A National Inpatient Sample Study

**DOI:** 10.3390/jcm14238354

**Published:** 2025-11-24

**Authors:** Ali L. Al-Batat, Tiberiu A. Pana, Mamas A. Mamas, Evangelos Kontopantelis, Phyo K. Myint

**Affiliations:** 1 Keele Cardiovascular Research Group, Centre for Prognosis Research, Institute for Primary Care and Health Sciences, Keele University, Stoke-on-Trent ST4 6QG, UK; albatat.ali@gmail.com (A.L.A.-B.); m.mamas@keele.ac.uk (M.A.M.); 2 Institute of Applied Health Sciences, School of Medicine, Medical Sciences and Nutrition, University of Aberdeen, Aberdeen AB25 2ZD, UK; tiberiu.pana@abdn.ac.uk; 3 Division of Informatics, Imaging and Data Sciences, The University of Manchester, Manchester M13 9NT, UK; e.kontopantelis@manchester.ac.uk

**Keywords:** ischaemic stroke, heart failure, revascularization, thrombolysis, thrombectomy

## Abstract

**Background/Objectives**: We aimed to determine sex and racial/ethnic disparities in associations between Acute Ischaemic stroke (AIS) with co-morbid heart failure (HF) and in-hospital outcomes, using the US National Inpatient Sample (NIS) in this retrospective observational study based on administrative data. **Methods**: AIS admissions from January 2016 to December 2019 were extracted from the NIS. Logistic regressions analysed relationships between HF and in-hospital mortality, length of stay >4 days, thrombectomy, thrombolysis, and complications at discharge adjusted for age and comorbidities. Additional models examined interactions between HF and sex, and HF and race/ethnicity. **Results**: Among 1,744,390 AIS hospitalizations, 16.36% had HF. 69.00% were White, 17.86% Black, 7.42% Hispanic, 2.79% Asian. No significant sex or racial/ethnic differences were found for in-hospital mortality. Patients with co-morbid HF had increased odds of complications at discharge in both sexes (odds ratio (95% confidence interval) 1.32 (1.27–1.37) in women and 1.21 (1.17–1.25) in men). HF (cf. no HF) showed stronger associations with increased complications at discharge in White (1.36 (1.32–1.40)) patients, compared to other racial/ethnic groups (Black 1.08 (1.03–1.13), Hispanic 1.10 (1.01–1.20), and Asian 1.13 (0.97–1.32)). HF was not significantly associated with thrombolysis in White patients (0.98 (0.95–1.02)) but was in Black (1.20 (1.13–1.28)), Hispanic (1.27 (1.14–1.40)), and Asian (1.36 (1.14–1.62)). Additionally controlling socioeconomic variables did not change the relationships except in Hispanic patients for complications, which has become insignificant (1.07 (0.98–1.17)). **Conclusions**: The association between HF and post-stroke outcomes differs by race/ethnicity and sex, even when adjusting for key predictors of outcomes. Further research is required to identify the drivers of these disparities.

## 1. Introduction

Heart failure (HF) is associated with acute ischaemic stroke (AIS) [1,2,3]. Pathophysiological mechanisms including thrombus formation due to atrial fibrillation (AF), and blood stasis due to left ventricular hypokinesia, particularly in patients with HF with reduced ejection fraction (HFrEF), contribute to the increased risk of AIS [4,5]. It has been shown that patients with AIS who have co-morbid HF have poorer outcomes compared to their counterparts without co-morbid HF [4,6]. While the previous literature has focused on the impact of HF in AIS patients, it has not differentiated between sex and race/ethnicity of the patients and the associated risk for each of these groups, highlighting a gap in the literature.

Furthermore, whilst revascularisation therapies are associated with significantly lower odds of in-hospital mortality in AIS with co-morbid HF [7], it is unclear whether these associations are influenced by sex or race/ethnicity. Importantly, previous reports from the NIS cohort suggest that racial/ethnic minority patients are less likely to receive revascularisation than White patients in general (16.6–18.6% rates of revascularisation therapies compared to 20.7–21.8% for White patients) [7].

Understanding sex and racial/ethnic disparities in AIS patients with co-morbid HF is crucial due to the high morbidity associated with both conditions. Identifying the underlying drivers of sex and racial/ethnic disparities can provide opportunities for targeted interventions to improve health outcomes and care equity in these populations. In this study, we therefore aim to determine the sex and racial/ethnic differences in the association between co-morbid HF and AIS treatments and in-hospital outcomes using NIS data. We hypothesised that co-morbid HF is associated with sex and racial/ethnic disparities in revascularisation treatments and in-hospital outcomes in AIS patients.

## 2. Materials and Methods

### 2.1. Data Source and Inclusion Criteria

NIS is the largest publicly available all-payer database in the United States. It represents a 20% stratified sample of all community hospital admissions in the US. This study was exempt from ethical review, as the data contained in NIS contained no patient identifiable information. Using the provided NIS strata, year of admission and sampling weights, the estimates determined are representative of ~97% of the US population [8].

All records from January 2016 to December 2019 with a primary diagnosis of ischaemic stroke (using all International Classification of Disease, 10th edition (ICD10) I63 code subtypes) were extracted from the NIS dataset [9].

**Figure 1:**
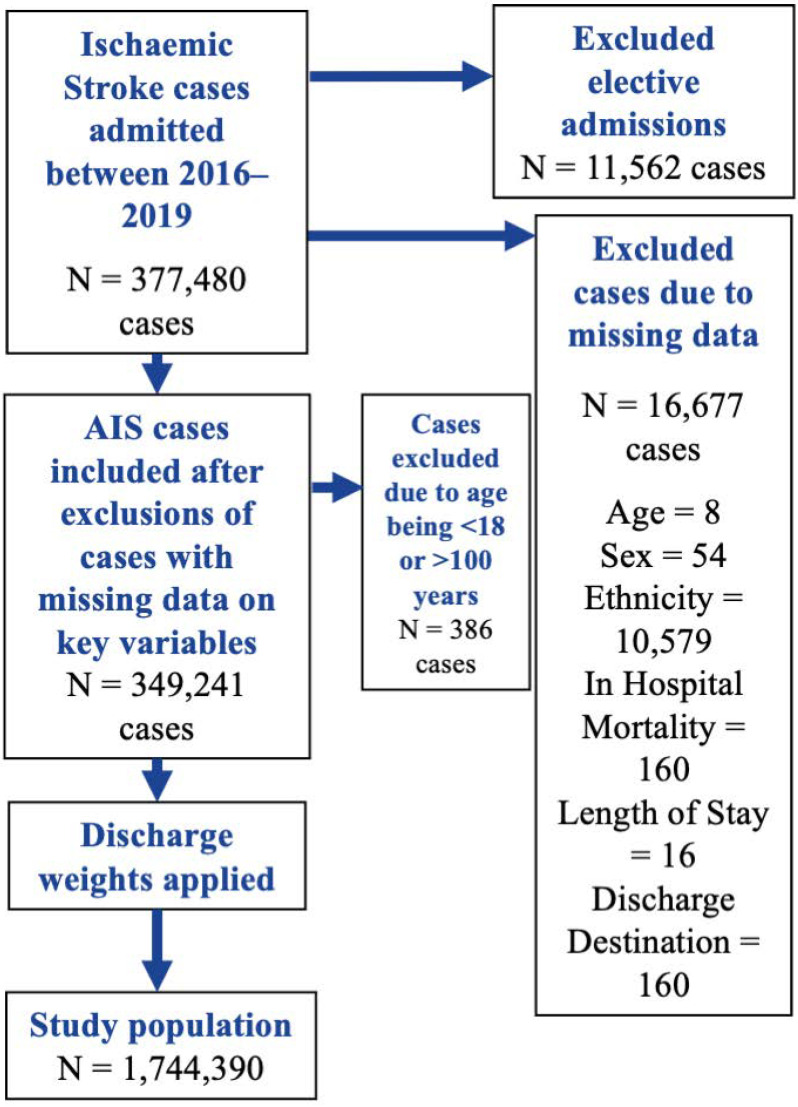
Flowchart outlining patient population. HF—Heart Failure, AIS—Acute Ischaemic Stroke.

A total of N = 377,480 cases with a primary diagnosis ischaemic stroke were identified and relevant data were extracted. Elective admissions were excluded to ensure only admissions due to acute stroke events were included in the study. A complete case approach was employed to exclude and remove all cases with missing data in key variables and outcomes (N = 16,677 cases) (Figure 1) prior to discharge weights being applied. Comorbidities were coded as binary indicators (present or absent) and therefore were considered to be complete. A total study population of N = 1,744,390 patients admitted with AIS between 2016 to 2019 was achieved.

### 2.2. Definition of Groups, Exposures, Confounders, and Outcomes

The NIS is derived from administrative hospital data; as such, sex and race/ethnicity are recorded at time of patient admission. Race/ethnicity in the NIS is based on a single variable that combines race and ethnicity as documented administratively by participating hospitals. The available categories are White, Black, Hispanic, Asian or Pacific Islander, Native American, and Other. Due to the small number of patients in the Native American and “Other” groups, these categories were combined for analysis.

The primary outcomes assessed in the study were inpatient mortality, prolonged hospital stay of more than 4 days, routine discharge, and receipt of Intravenous Thrombolysis (IVT) (which includes any thrombolytic medicine) and Endovascular Thrombectomy (ET). NIS variable coding was used to determine vital status on discharge [10]. Prolonged hospital stay was defined as a hospitalisation of more than 4 days, as the median length of stay in non-HF patients with AIS was 3 days. This was in line with clinical opinion and previous studies [8,11,12].

Routine discharge was defined by using the discharge destination variable in the dataset [13]. Patients discharged against medical advice or discharged to unknown destinations were excluded from the analysis. The discharge destination was then further grouped into routine discharge (patients who were discharged home) and other discharge (this includes patients who were discharged to: “home healthcare”, “short-term hospital”, “other facilities including intermediate care and skilled nursing home”). The discharge destination has previously been correlated strongly with post-stroke functional outcomes, demonstrating a high predictive value for unfavourable outcomes defined by the modified Rankin scale. Therefore, a previously validated method of using the discharge destination to determine complications at discharge was employed [7,14]. Routine discharge was classed as none to minimal complications at discharge, while other discharge was classed as moderate to severe complications at discharge.

Procedural ICD-10 codes were used to identify patients that underwent IVT (3E03317; 3E04317; 3E05317; 3E06317; Z9282) or ET (03CG3ZZ; 03CG4ZZ; 03CK3Z7).

HF was the primary exposure of interest. Patients with the respective ICD-10 codes to encompass all HF subtypes were identified (I50.1; I50.20; I50.21; I50.22; I50.23; I50.30; I50.31; I50.32; I50.33; I50.40; I50.41; I50.42; I50.43; I50.810; I50.811; I50.812; I50.813; I50.814; I50.82; I50.83; I50.84; I50.89; I50.9; I09.81; I11.0; I13.0; I13.2) and subdivided into groups based on their sex and race/ethnicity.

### 2.3. Statistical Analysis

All analyses were conducted with Stata 16 SE (StataCorp, TX, USA) statistical software. A 5% alpha level was used for all analyses, which were completed following HCUP guidelines [15]. Discharge weights were incorporated using the Stata svy command with the NIS hospital number as the primary sampling unit and stratification by admission year and NIS stratum for all analytical models. Prior to any analysis and application of discharge weights, any patient with missing data in key outcomes and variables were excluded and removed from the dataset. All analyses and reporting followed the Strengthening the Reporting of Observational Studies in Epidemiology (STROBE) guidelines for cohort studies [16].

### 2.4. Descriptive Statistics

Patient characteristics were compared across the 2 exposure categories (HF and no HF) using the Pearson’s *t*-test and the Kruskal–Wallis tests if normality was established or not, respectively.

### 2.5. Primary Analysis

All analytical models used the no HF group within each patient subgroup as the reference category and adjusted for race/ethnicity, sex, and additional covariates (see the following section). To determine the in-hospital outcomes or the use of IVT or ET in AIS patients with or without HF, multivariable logistic regression modelling was performed. Additionally, to fully capture the relationship between the key exposure with race/ethnicity and sex, two additional models were executed with an appropriate interaction term included in each (sex with HF and race/ethnicity with HF).

### 2.6. Sensitivity Analyses

A sensitivity analysis with two additional models was executed with appropriate interaction terms (sex with HF and race/ethnicity with HF) to estimate predicted probabilities and absolute risk.

Sensitivity analyses were conducted to assess any potential effects of including socioeconomic covariates. Therefore, the models described in Section 2.5 were executed with socioeconomic indicators such as insurance payer, zip code income quartile, and hospital region as covariates.

Further sensitivity analyses were conducted to assess the potential effect modification by age. Two additional models were executed with interaction terms between HF and age group (65<, 65–80, >80 years) within sex- and race/ethnicity-stratified analyses to explore the relationships between HF and study outcomes varied across age strata.

### 2.7. Adjusting Covariates

All models were adjusted for age, race/ethnicity, atrial fibrillation, coronary heart disease, deep vein thrombosis, infective endocarditis, peripheral vascular disease, previous valve surgery, ventricular tachycardia, venous thromboembolism, alcohol, anaemia, use of anticoagulation, use of antiplatelets, arthritis, bleeding, chronic lung disease, dementia, diabetes, drug abuse, epilepsy, HIV, hypertension, hypotension, lipid disorders, liver disorders, malignancy, malnutrition smoking, non-rheumatic heart disease, obesity, pneumonia, previous coronary artery bypass graft, psychiatric disorders, renal disorders, respiratory failure, rheumatic heart disease, sepsis, thyroid disorders, and viral hepatitis (Supplement S1. Table S1). Multicollinearity among comorbidities was assessed using variance inflation factors (VIF), with all values < 5 (mean VIF = 1.11), indicating no significant collinearity.

## 3. Results

### 3.1. Descriptive Statistics

A sample representative of 1,744,390 patients was included in this study. 69% were White, 17.9% Black, 7.4% Hispanic, 2.8% Asian or Pacific Islander and 2.9% were identified as the “Other” racial/ethnic group (Table 1).

The median age was 71 years for the whole cohort. The median age of the HF group was higher than the no HF group (76 and 71 years, respectively) (Table 1). This trend was consistent across all racial/ethnic groups with HF included in the study (Supplement S2. Table S2).

A higher proportion of HF patients had comorbidities (Table 1), such as Coronary Heart Disease (53.9% of patients with HF had CHD vs. 23.7% of patients without HF), Chronic Lung Disease (26.4 of patients with HF had Chronic Lung Disease vs. 14.4% of patients without HF), Hypertension (92.8% of patients with HF had hypertension vs. 84.8% of patients without), Diabetes Mellitus (46.7% of patients with HF had diabetes vs. 37.6% of patients without).

### 3.2. Primary Analysis

#### 3.2.1. Association Between Revascularisation Therapy and Race/Ethnicity and Sex

**Figure 2:**
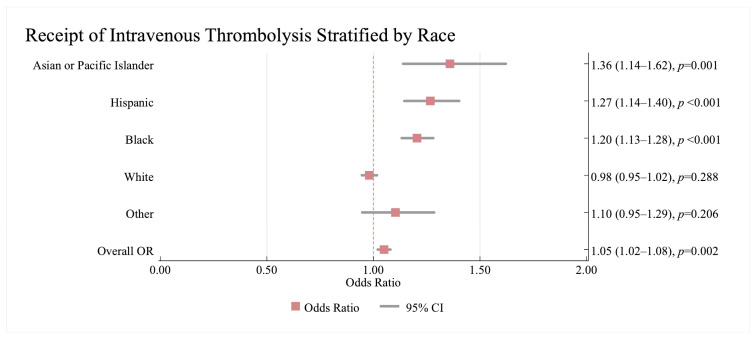
The results of the multivariate analysis showing of the association between HF and receiving Intravenous thrombolysis stratified by race in acute ischaemic stroke patients with heart failure. Results are reported as (odds ratio (95% confidence interval)). The no HF category is used as a reference. All models were adjusted for age, sex, pre-existing cardiovascular and non-cardiovascular conditions on admission. Statistically significant results are reported with *p* < 0.05. OR—Odds Ratio, CI—Confidence Interval, IVT—Intravenous Thrombolysis.

The multivariate logistic regression model shows a significant association between HF and receipt of IVT in AIS patients (Odds Ratio (OR) = 1.05; 95% Confidence Interval (CI): 1.02, 1.08, *p* = 0.002) (Figure 2).

Our results showed a significant interaction between race/ethnicity and the association between HF and IVT (*p* < 0.001). While the association between HF and receipt of IVT in White patients was not significant (0.98; 95%CI:0.95, 1.02, *p* = 0.288), HF was associated with higher odds of receiving IVT in all other racial/ethnic minority patients: Black: 1.20; 95%CI: 1.13, 1.28, Hispanic: 1.27; 95%CI: 1.14, 1.40, and Asian: 1.36; 95%CI: 1.14, 1.62. Black patients with HF had a 20% higher chance of receiving IVT compared to their counterparts without HF as a co-morbid condition in AIS, while Hispanic patients with HF had a 27% higher chance of receiving IVT (Figure 2).

We found no significant interaction between men and women with HF for receipt of IVT. (*p* = 0.231).

Co-morbid HF was significantly associated with increased odds of receiving ET, compared to no HF (1.16; 95%CI: 1.09, 1.23, *p* < 0.001)

**Figure 3:**
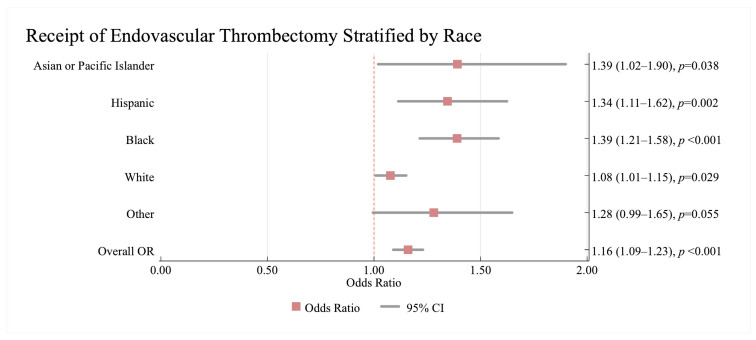
The results of the multivariate analysis showing of the association between HF and receiving Endovascular Thrombectomy stratified by race/ethnicity in acute ischaemic stroke patients. Results are reported as (odds ratio (95% confidence interval)). The no HF category is used as a reference. All models were adjusted for age, sex, pre-existing cardiovascular and non-cardiovascular conditions on admission. Statistically significant results are reported with *p* < 0.05. OR—Odds Ratio, CI—Confidence Interval, ET—Endovascular Thrombectomy.

We also show a significant interaction between HF and race/ethnicity for receipt of ET (*p* = 0.001). While HF was associated with significant higher odds of receipt of ET in all racial/ethnic groups (using the no HF category within each subgroup as the reference), this association was stronger in Black (1.39; 95%CI: 1.21, 1.58) and Hispanic (1.34; 95%CI: 1.11, 1.62) than in White patients (1.08; 95%CI: 1.01, 1.15) (Figure 3).

Similarly to IVT, the interaction between men and women with co-morbid HF was not significant for ET (*p* = 0.932).

#### 3.2.2. Association Between In-Hospital Mortality and Race/Ethnicity and Sex

Our results show no significant association between HF and in-hospital mortality (1.050; 95%CI: 0.995, 1.109, *p* = 0.073), therefore no interaction models were constructed for further analysis (Supplement S2, Figure S1).

#### 3.2.3. Association Between Complications at Discharge and Race/Ethnicity and Sex

**Figure 4:**
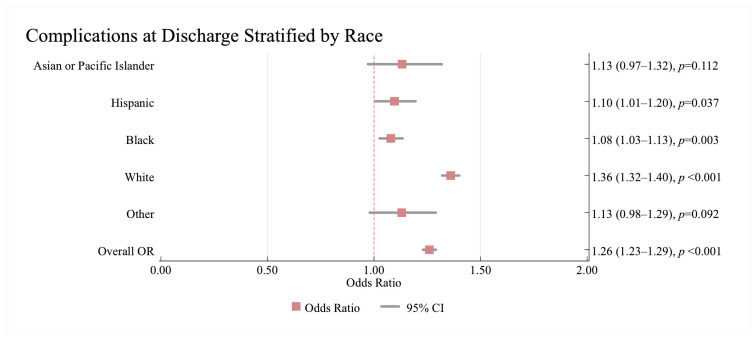
The results of the multivariate analysis showing of the association between HF and complications at discharge stratified by race/ethnicity in acute ischaemic stroke patients with heart failure. Results are reported as (odds ratio (95% confidence interval)). The no HF category is used as a reference. All models were adjusted for age, sex, pre-existing cardiovascular and non-cardiovascular conditions on admission. Statistically significant results are reported with *p* < 0.05. OR—Odds Ratio, CI—Confidence Interval.

As a group, HF was associated with significantly increased odds of complications at discharge (1.26; 95%CI: 1.23, 1.29, *p* < 0.001). However, there was a significant interaction between race/ethnicity and the association between HF and complications at discharge (*p* < 0.001). While the association between HF and complications at discharge for Asian or Pacific Islander patients was not significant (1.13; 95%CI: 0.97, 1.32), Black, Hispanic, and White patients with HF displayed a significant association between complications at discharge. Black and Hispanic patients with HF had a significant association with complications at discharge (Black 1.08; 95%CI: 1.03, 1.13, *p* = 0.003, Hispanic 1.10; 95%CI: 1.01, 1.20, *p* = 0.037), whereas White patients with HF had a stronger association with having complications at discharge (1.36; 95%CI: 1.32, 1.40, *p* < 0.001) (Figure 4).

**Figure 5:**
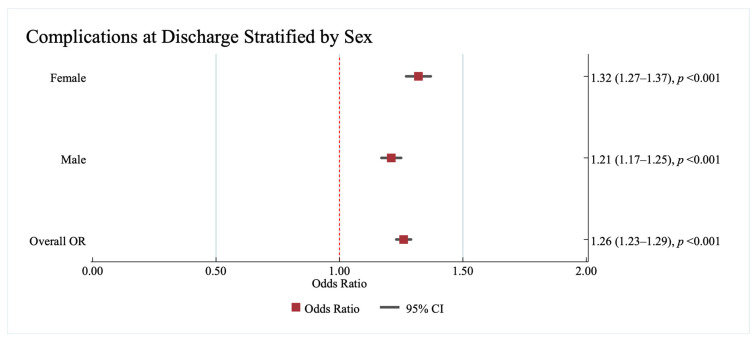
The results of the multivariate analysis showing of the association between HF and complications at discharge stratified by sex in acute ischaemic stroke patients with heart failure. Results are reported as (odds ratio (95% confidence interval)). The no HF category is used as a reference. All models were adjusted for age, race/ethnicity, pre-existing cardiovascular and non-cardiovascular conditions on admission. Statistically significant results are reported with *p* < 0.05. OR—Odds Ratio, CI—Confidence Interval.

Figure 5 details the results of the multivariate logistic regression analysing the association between sex and complications at discharge with the no HF group as the reference category. Our results show a significant interaction between sex and HF for complications at discharge (*p* < 0.001). Women with HF had 32% increased odds of developing complications at discharge (1.32; 95%CI: 1.27, 1.37, *p* < 0.001), while men had 21% odds of developing complications at discharge (1.21; 95%CI: 1.17, 1.25, *p* < 0.001) compared to their counterparts without HF as a co-morbid condition in AIS.

#### 3.2.4. Association Between Median Length of Stay and Race/Ethnicity and Sex

The results of our multivariate logistic regression found a significant association between HF and increased median LoS > 4 days (1.29; 95%CI: 1.26, 1.32) (Supplement S2, Figure S1). There was no significant interaction between race/ethnicity and LoS > 4 days (*p* = 0.80) and sex (*p* = 0.80).

### 3.3. Sensitivity Analyses

#### 3.3.1. Analyses Generating Absolute Risk Differences for Outcomes and Treatments

##### The Association Between Revascularization Therapy and Race/Ethnicity and Sex

Our sensitivity analysis shows that the absolute risk difference for the association between HF and receipt of IVT in White patients was not significant (Absolute Risk Difference (ARD) = −0.22; 95%CI: −0.62, 0.18, *p* = 0.286). Patients with HF had higher odds of receiving IVT in all other racial/ethnic minority patients: Black ARD = 1.86; 95%CI: 1.22, 2.50, Hispanic ARD = 2.81; 95%CI: 1.54, 4.09, and Asian ARD = 3.54; 95%CI: 1.33, 5.74 (Supplement S2 Table S4).

Patients with HF had significantly higher odds of the receipt of ET in all racial/ethnic groups. However, this association was stronger in Black (ARD = 0.77; 95%CI: 0.43, 1.12) and Hispanic (ARD = 0.93; 95%CI: 0.23, 1.30) than in White patients (ARD = 0.19; 95%CI: 0.02, 0.36) (Supplement S2 Table S5).

##### The Association Between Complications at Discharge and Race/Ethnicity and Sex

The sensitivity analysis did not find any change in the significance for the association between HF and complications at discharge for any patient cohort.

Black and Hispanic patients with HF had a significant association with complications at discharge (Black ARD = 1.47; 95%CI: 0.51, 2.45, Hispanic ARD = 1.83; 95%CI: 0.12, 3.53), whereas White patients with HF had the strongest association with having complications at discharge (ARD = 5.84; 95%CI: 5.29, 6.40) (Supplement S2 Table S6).

Both men and women with co-morbid HF had a stronger association with complications at discharge than their counterparts without HF (men: ARD = 3.92; 95% CI: 3.33, 4.58, women: ARD = 4.98; 95%CI: 4.35, 5.60).

#### 3.3.2. Analysis Adjusted for Socioeconomic Indicators

Socioeconomic adjustments did not cause any material difference in the significance of all interactions reported.

However, the inclusion of socioeconomic covariates led to some associations not being statistically significant any more, namely “Other” patients receiving IVT (1.11; 95%CI: 0.95, 1.29, *p* = 0.129), ET (1.29; 95%CI: 1.00, 1.65, *p* = 0.050), as well as complications for Hispanic patients (1.07; 95%CI: 0.98, 1.17, *p* = 0.107).

#### 3.3.3. Age Subgroup Analysis

**Figure 6:**
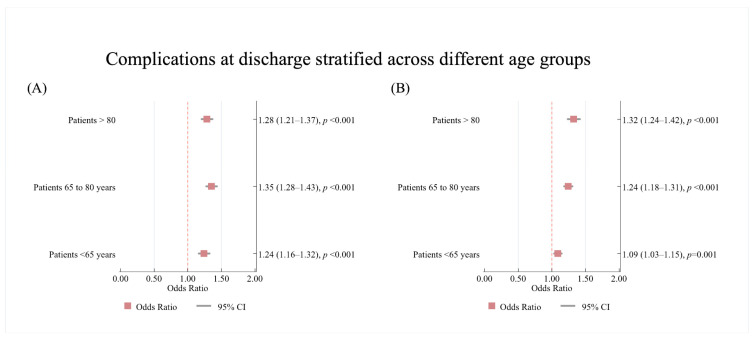
The results of the multivariate analysis showing of the association between HF and complications at discharge stratified by age groups in (**A**) women and (**B**) men with acute ischaemic stroke patients with heart failure. Results are reported as (odds ratio (95% confidence interval)). The no HF category was used as the reference category. All models were adjusted for age, race, pre-existing cardiovascular and non-cardiovascular conditions on admission. Statistically significant results reported with *p* < 0.05. OR—Odds Ratio, CI—Confidence Interval.

The results of our sensitivity analysis examining whether treatment outcomes varied by age group (<65, 65–80, and >80 years) found that the interaction between HF and race/ethnicity was not statistically significant for IVT (*p* = 0.371), ET (*p* = 0.288) or complications at discharge (*p* = 0.983). However, the interaction between HF and sex was significant for complications at discharge (*p* = 0.028). Among women with co-morbid HF, those aged < 65 had 24% higher odds of complications at discharge compared to those without HF (1.24; 95%CI: 1.16, 1.32, *p* < 0.001). The odds of complications at discharge increased for women aged between 65 and 80 years (1.35; 95%CI: 1.28, 1.43, *p* < 0.001) before decreasing for women > 80 years (1.28; 95%CI: 1.21, 1.37, *p* < 0.001) (Figure 6A).

A similar trend was observed in men with co-morbid HF, who had higher odds of complications at discharge than their counterparts without HF across all age groups (<65 years: 1.09; 85%CI: 1.03, 1.15, *p* = 0.001; 65–80 years: 1.24; 1.18, 1.31, *p* < 0.001; >80 years: 1.32; 95%CI: 1.24, 1.42, *p* < 0.001) (Figure 6B).

## 4. Discussion

To the best of our knowledge, this is the first study to examine the sex and racial/ethnic disparities on the association between co-morbid HF on treatment strategies and clinical outcomes in patients with AIS. Using the US Nationally representative sample with a sample population of 1,744,390 AIS patients admitted between 2016 and 2019, we have found the association between HF and receipt of IVT and ET to be significantly stronger among Black patients compared to White patients. The association between HF and complications at discharge was stronger among White patients than with Black patients. There were no significant associations between HF and in-hospital mortality.

Prior studies have consistently shown that racial/ethnic minority patients have lower IVT rates when compared to White patients in the general AIS population. However, whether these disparities extend to patients with co-morbid HF has not been investigated [17]. We have identified a significant association between HF and higher odds of receiving IVT in Black, Hispanic, and Asian and Pacific Island patients. White patients with HF were significantly older than all other racial/ethnic minority groups (Supplement S2 Table S3) (79 years vs. 67 for Black vs. 73 for Hispanic vs. 76 for Asian/Pacific Islander vs. 72 for Other patients with HF), which may have influenced the choice of treatment modalities. While we adjusted for age, previous work has indeed demonstrated that increasing age can lead to an increase in door-to-needle time which could contributes to lower odds of receiving IVT, which may have confounded our results [18]. Nevertheless, our results highlight a potential difference in IVT accessibility and reinforce the need for continued research into healthcare equity and AIS aetiology.

As with the IVT receipt, previous work has also reported lower rates of ET among racial/ethnic minority patients with AIS [17]. In contrast, we observed a significant association between HF and receiving ET in all racial/ethnic groups. This association was notably stronger in Black, Hispanic, and Asian, and Pacific Islander patients when compared to White patients. This is unexpected because both Black and Hispanic patients had a lower prevalence of AF (Black 31.35% vs. Hispanic 42.46% vs. White 53.8%) but higher rates of Diabetes Mellitus (Black 53.77% vs. Hispanic 58.18% vs. White 42.91%) and Hypertension (Black 95.35% vs. Hispanic 94.83% vs. White 91.75%) (Supplement S2, Table S3), with both conditions associated with small vessel strokes [19]. The findings suggest additional factors such as diagnostic difficulties in ascertaining stroke subtypes, healthcare access, or unmeasured confounders may contribute to our observed disparities [20].

Similarly, other works have shown that socioeconomic factors such as hospital region, zip income quartile, and insurance coverage can influence access to reperfusion therapies and post-stroke outcomes [17,21]. The change in significance for Hispanic patients and the “Other” patient groups after adjusting for these factors may reflect regional differences in healthcare infrastructure and delivery. These findings therefore suggest that structural and system-level factors may contribute to the observed disparities in treatments and outcomes among patients with AIS and HF.

Beyond demographic disparities in revascularisation treatment, differences in HF phenotypes across racial/ethnic groups may contribute to our results. Previous work suggests White patients have higher lifetime risks of HF with preserved ejection fraction (HFpEF) than other racial/ethnic groups. Black patients with HFpEF were also found to be younger than their White counterparts [22]. A small study examining the AIS outcomes in HFpEF and HFrEF in 108 patients in China found no significant difference in reperfusion treatment between the HF phenotypes. However, HFpEF patients had more unfavourable outcomes compared to HFrEF patients with ischaemic stroke [23]. This highlights the need for more large-scale granular studies to investigate the effects of HF phenotypes on AIS outcomes and different racial/ethnic groups. This could inform strategies for improved secondary prevention and healthcare equity between racial/ethnic minority groups.

Contrasting existing studies focusing on racial disparities in AIS [24], our results show that HF was associated with more complications at discharge in White patients when compared to Black patients. Previous works suggest racial/ethnic minority patients suffered from more severe strokes than White patients. The main drivers found to contribute to stroke severity were higher rates of smoking, older age, and comorbidities [25]. A possible explanation for our results could be that among HF patients, White patients may have a more severe phenotype [26]. This is consistent with the significantly older age of White patients with HF when compared to other racial/ethnic minority groups. Whilst we adjusted for differences in comorbidities, we were unable to adjust our data for differences in stroke severity or frailty which may be more common in older patients [23,26].

Age did not materially influence the main associations in our analysis. The supplementary age-stratified analysis showed that patient age groups had no significant effect for different racial/ethnic groups across complications at discharge, IVT, and ET receipt. A modest interaction was observed between men and women and age group, which suggests limited age-related variation in sex specific outcomes.

We also identified significant sex disparities in the associations between HF and complications at discharge. Female patients were more likely to be discharged to a secondary care facility than male patients. This is consistent with previous work using the Danish stroke registry [27]. A possible cause of this might be that women may have presented with a more severe phenotype or were more likely to be living alone or widowed at time of AIS [28].

### Strengths and Limitations

Our study has several strengths, such as our study population of 1,744,390 patients admitted for AIS across the US between 2015 and 2019. Our results therefore reflect the mainstay management of AIS across the US, including outcomes such as the hospital length of stay.

This study also has several limitations. First, we used administrative data and ICD-10 codes to define AIS and HF, which may introduce misclassification bias for both predictor and outcomes; it also limited our ability to distinguish between stroke subtypes and HF phenotypes (HFrEF vs. HFpEF). Consequently, we could not adjust for the different treatment pathways or outcome differences associated with these subtypes. Additionally, race/ethnicity in the NIS reflects social and administrative classifications with variable heterogeneity (e.g., Asians may include individuals of East Asian, South Asian or Pacific Islander descent), which could be influenced by social and structural factors rather than inherent biological differences.

While the use of discharge destination as a substitute for the modified Rankin scale is a validated method of classifying complications at discharge, the NIS does not capture stroke severity or other clinical indicators that may influence these decisions. Consequently, discharge destination is not always determined by functional status alone. Institutional factors such as inpatient rehabilitation capabilities or insurance cover may also determine discharge destination and thus confound our results.

Our analysis included only hospitalised patients, excluding out-of-hospital stroke deaths, which may introduce selection bias. Nevertheless, the paper focused on disparities in treatment received and outcomes by sex and race/ethnicity and out of hospital deaths fell outside the scope of our work. Although our data was nationally representative, some racial/ethnic minority groups may be underrepresented (e.g., not having access to healthcare), which can further affect the generalisability of our findings.

Finally, residual confounding from adjusted covariates or unknown confounders may remain despite adjustments. Our data did not include stroke severity, pre-stroke functional status, onset-to-door and door-to-needle times. The omission of these confounders can result in additional unobserved confounding. Additionally, temporal changes to practices or treatment strategies between 2016 and 2019 may have introduced time-varying confounding. Further research with more granular clinical data is therefore required to validate our results.

## 5. Conclusions

In conclusion, using the NIS, which is the largest all-payer database of US inpatient admissions, we have demonstrated that the association between HF and adverse outcomes after AIS differs by race/ethnicity and sex. While race/ethnicity in the NIS mainly reflect social and structural factors, it may also capture biological and clinical differences across populations. Studies utilising more granular clinical data are required to clarify the mechanisms driving these disparities and to inform interventions that can promote equity in stroke care for HF patients.

## Figures and Tables

**Table 1:** Characteristics on admission, stratified by co-existent HF, HF only or no HF. HF—Heart Failure.

	Total	No HF	HF	*p* Value
N	1,744,390	1,459,035	285,355	
Age, y, median (IQR),	71.0 (61.0–82.0)	71.0 (60.0–81.0)	76.0 (65.0–85.0)	<0.001
Length of stay, d, median (IQR)	3.0 (2.0–6.0)	3.0 (2.0–5.0)	4.0 (3.0–7.0)	<0.001
Mortality, n, (%)	61,295 (3.5)	42,980 (3.0)	18,315 (6.4)	<0.001
Sex, n, (%)				
Female	876,100 (50.2)	731,590 (50.1)	144,510 (50.6)	0.029
Male	868,290 (49.8)	727,445 (49.9)	140,845 (49.4)	0.029
Race/Ethnicity, n, (%)				
White	1,203,595 (69.0)	1,012,210 (69.4)	191,385 (67.1)	<0.001
Black	311,600 (17.9)	249,985 (17.1)	61,615 (21.6)	<0.001
Hispanic	129,460 (7.4)	111,000 (7.6)	18,460 (6.5)	<0.001
Asian or Pacific Islander	48,650 (2.8)	42,665 (2.9)	5985 (2.1)	<0.001
Other	51,085 (2.9)	43,175 (3.0)	7910 (2.8)	<0.001
Comorbidities, n, (%)				
Atrial Fibrillation	442,345 (25.36)	305,625 (20.95)	136,720 (47.91)	<0.001
Coronary Heart Disease	499,795 (28.65)	346,050 (23.72)	153,745 (53.88)	<0.001
Deep Vein Thrombosis	25,160 (1.44)	19,840 (1.36)	5320 (1.86)	<0.001
Infective Endocarditis	6110 (0.35)	4450 (0.30)	1660 (0.58)	<0.001
Peripheral vascular disease	146,915 (8.42)	115,170 (7.89)	31,745 (11.12)	<0.001
Previous Valve Surgery	32,920 (1.89)	21,420 (1.47)	11,500 (4.03)	<0.001
Ventricular Tachycardia	27,365 (1.57)	15,645 (1.07)	11,720 (4.11)	<0.001
Venous Thromboembolism	100,805 (5.78)	78,840 (5.40)	21,965 (7.70)	<0.001
Alcoholism	77,050 (4.42)	66,960 (4.59)	10,090 (3.54)	<0.001
Anaemia	240,785 (13.80)	177,930 (12.20)	62,855 (22.03)	<0.001
Use of Anticoagulant(s)	176,290 (10.11)	124,365 (8.52)	51,925 (18.20)	<0.001
Use of antiplatelet(s)	504,935 (28.95)	417,395 (28.61)	87,540 (30.68)	<0.001
Arthritis	195,385 (11.20)	157,950 (10.83)	37,435 (13.12)	<0.001
Bleeding disorder	62,290 (3.57)	47,135 (3.23)	15,155 (5.31)	<0.001
Chronic lung disease	285,390 (16.36)	210,160 (14.40)	75,230 (26.36)	<0.001
Dementia	205,225 (11.76)	162,610 (11.15)	42,615 (14.93)	<0.001
Diabetes mellitus	681,665 (39.08)	548,365 (37.58)	133,300 (46.71)	<0.001
Drug abuse	65,110 (3.73)	55,215 (3.78)	9895 (3.47)	<0.001
Epilepsy	74,145 (4.25)	61,060 (4.18)	13,085 (4.59)	<0.001
Human Immunodeficiency virus	3565 (0.20)	3035 (0.21)	530 (0.19)	0.268
Hypertension	1,501,600 (86.08)	1,236,840 (84.77)	264,760 (92.78)	<0.001
Hypotension	62,690 (3.59)	46,375 (3.18)	16,315 (5.72)	<0.001
Hyperlipidaemia	1,055,995 (60.54)	880,600 (60.35)	175,395 (61.47)	<0.001
Liver disease	28,410 (1.63)	21,610 (1.48)	6800 (2.38)	<0.001
Malignancy	79,295 (4.55)	66,615 (4.57)	12,680 (4.44)	0.191
Malnutrition	128,770 (7.38)	101,860 (6.98)	26,910 (9.43)	<0.001
Smoking cigarettes	331,265 (18.99)	291,250 (19.96)	40,015 (14.02)	<0.001
Non-rheumatic valve disease	95,605 (5.48)	67,320 (4.61)	28,285 (9.91)	<0.001
Obesity	244,100 (13.99)	197,595 (13.54)	46,505 (16.30)	<0.001
Pneumonia	98,410 (5.64)	68,160 (4.67)	30,250 (10.60)	<0.001
Previous coronary artery bypass graft	114,705 (6.58)	78,445 (5.38)	36,260 (12.71)	<0.001
Psychiatric disease	353,320 (20.25)	294,905 (20.21)	58,415 (20.47)	0.180
Renal disease	442,575 (25.37)	314,180 (21.53)	128,395 (44.99)	<0.001
Respiratory failure	118,925 (6.82)	76,205 (5.22)	42,720 (14.97)	<0.001
Rheumatic heart disease	52,285 (3.00)	34,285 (2.35)	18,000 (6.31)	<0.001
Sepsis	26,950 (1.54)	18,295 (1.25)	8655 (3.03)	<0.001
Thyroid disease	285,940 (16.39)	231,995 (15.90)	53,945 (18.90)	<0.001
Viral hepatitis	16,075 (0.92)	13,245 (0.91)	2830 (0.99)	0.059
Thrombolysis	220,830 (12.66)	184,630 (12.65)	36,200 (12.69)	0.837
Endovascular thrombectomy	45,690 (2.62)	34,790 (2.38)	10,900 (3.82)	<0.001
Patient Admission year, n, (%)				
2016	438,825 (25.2)	372,205 (25.5)	66,620 (23.4)	<0.001
2017	461,005 (26.4)	386,720 (26.5)	74,285 (26.0)	<0.001
2018	327,720 (18.8)	272,665 (18.7)	55,055 (19.3)	<0.001
2019	516,840 (29.6)	427,445 (29.3)	89,395 (31.3)	<0.001

## Data Availability

The data supporting the findings of this study are available from the corresponding author upon reasonable request.

## Supplementary Materials

The following supporting information can be downloaded at https://www.mdpi.com/article/10.3390/jcm14238354/s1, Supplement S1 Table S1: List of the International Classification of Diseases 10th Edition (ICD10) codes utilised to identify covariates; S1: Strobe statement; Supplement S2 Figure S1: The results of the multivariate logistic regression with no interaction terms. Patients without HF are used as reference. Results are reported as (odds ratio (95% confidence interval). All models were adjusted for age, race/ethnicity, pre-existing cardiovascular and non-cardiovascular conditions on admission. Statistically significant results are reported with *p* < 0.05. Odds ratios and confidence intervals are reported to three decimal places for mortality, where necessary to reflect statistical significance; all other outcomes are reported to two decimal places. AIS—Acute Ischaemic Stroke, LoS—Length of Stay, OR—Odds Ratio, CI—Confidence Interval; Supplement S2 Table S2: Descriptive statistics of AIS patients with HF stratified by sex. HF—Heart Failure; Supplement S2 Table S3: Descriptive statistics of AIS patients with HF stratified by racial/ethnic group. HF—Heart Failure; Supplement S2 Table S4: The results of the multivariate analysis showing of the association between HF and receipt of Intravenous Thrombolysis stratified by race/ethnicity in acute ischaemic stroke patients with heart failure. Results are reported as (Predicted Probabilities (95% confidence interval (CI))) and (Absolute risk difference (95% CI)). The no HF category is used as a reference. All models were adjusted for age, sex, pre-existing cardiovascular and non-cardiovascular conditions on admission. Statistically significant results are reported with *p* < 0.05. OR—Odds Ratio, CI—Confidence Interval; Supplement S2 Table S5: The results of the multivariate analysis showing of the association between HF and receipt of Endovascular Thrombectomy stratified by race/ethnicity in acute ischaemic stroke patients with heart failure. Results are reported as (Predicted Probabilities (95% confidence interval (CI))) and (Absolute risk difference (95% CI)). The no HF category is used as a reference. All models were adjusted for age, sex, pre-existing cardiovascular and non-cardiovascular conditions on admission. Statistically significant results are reported with *p* < 0.05. OR—Odds Ratio, CI—Confidence Interval; Supplement S2 Table S6: The results of the multivariate analysis showing of the association between HF and Complications at discharge stratified by race/ethnicity and sex in acute ischaemic stroke patients with heart failure. Results are reported as (Predicted Probabilities (95% confidence interval (CI))) and (Absolute risk difference (95% CI)). The no HF category is used as a reference. All models were adjusted for age, pre-existing cardiovascular and non-cardiovascular conditions on admission. Statistically significant results are reported with *p* < 0.05. OR—Odds Ratio, CI—Confidence Interval.

## Abbreviations

The following abbreviations are used in this manuscript:

HF	Heart Failure
AIS	Acute Ischaemic Stroke
AF	Atrial Fibrillation
HFrEF	Heart Failure with reduced ejection fraction
NIS	National Inpatient Sample
ICD10	International Classification of Disease, 10th edition
IVT	Intravenous Thrombolysis
ET	Endovascular Thrombectomy
LOS	Length of Stay
HCUP	Healthcare Cost and Utilisation Project
STROBE	Strengthening the Reporting of Observational Studies in Epidemiology
HFpEF	Heart failure with preserved ejection fraction
