# Supplementary material for: Sex and Ethnic Disparities in Stroke Revascularisation Treatments and Post-Stroke Outcomes in Patients with Heart Failure: A National Inpatient Sample Study
Source: J Clin Med. 2025 Nov 24;14(23):8354. doi: 10.3390/jcm14238354 (PMC12692724; doi:10.3390/jcm14238354)
Supplement: Supplementary file 1 [file jcm-14-08354-s001.zip › Supplement S2.pdf]

Supplement S2

Supplementary tables

Table S2. Descriptive statistics of AIS patients with HF stratified by sex. HF—Heart Failure.

	Total	Male	Female	P Value
N	285355	140845	144510	
Age, y, median (IQR)	76.0 (65.0-85.0)	72.0 (62.0-82.0)	80.0 (69.0-87.0)	<0.001
Length of stay, d, median, (IQR)	4.00 (3.0-7.0)	4.00 (3.0-7.0)	4.0 (3.0-7.0)	0.006
Mortality, n, (%)	18315 (6.4)	8440 (6.0)	9875 (6.8)	<0.001
Ethnicity, n, (%)				
White	191385 (67.1)	93540 (66.4)	97845 (67.7)	<0.001
Black	61615 (21.6)	30205 (21.5)	31410 (21.7)	<0.001
Hispanic	18460 (6.5)	9990 (7.1)	8470 (5.9)	<0.001
Asian or Pacific Islander	5985 (2.1)	3040 (2.2)	2945 (2.0)	<0.001
Other	7910 (2.8)	4070 (2.9)	3840 (2.7)	<0.001
Comorbidities, n, (%)				
Atrial Fibrillation	136720 (47.91)	63725 (45.24)	72995 (50.51)	<0.001
Coronary Heart Disease	153745 (53.88)	84325 (59.87)	69420 (48.04)	<0.001
Deep Vein Thrombosis	5320 (1.86)	2770 (1.97)	2550 (1.76)	0.078
Infective Endocarditis	1660 (0.58)	830 (0.59)	830 (0.57)	0.813
Peripheral vascular disease	31745 (11.12)	16420 (11.66)	15325 (10.60)	<0.001
Previous Valve Surgery	11500 (4.03)	5960 (4.23)	5540 (3.83)	0.017
Ventricular Tachycardia	11720 (4.11)	7665 (5.44)	4055 (2.81)	<0.001
Venous Thromboembolism	21965 (7.70)	10060 (7.14)	11905 (8.24)	<0.001
Alcoholism	10090 (3.54)	8335 (5.92)	1755 (1.21)	<0.001
Anemia	62855 (22.03)	27875 (19.79)	34980 (24.21)	<0.001
Use of Anticoagulant(s)	51925 (18.20)	26090 (18.52)	25835 (17.88)	0.045
Use of antiplatelet(s)	87540 (30.68)	44470 (31.57)	43070 (29.80)	<0.001
Arthritis	37435 (13.12)	13170 (9.35)	24265 (16.79)	<0.001
Bleeding disorder	15155 (5.31)	8150 (5.79)	7005 (4.85)	<0.001
Chronic lung disease	75230 (26.36)	34270 (24.33)	40960 (28.34)	<0.001
Dementia	42615 (14.93)	16135 (11.46)	26480 (18.32)	<0.001
Diabetes mellitus	133300 (46.71)	68535 (48.66)	64765 (44.82)	<0.001
Drug abuse	9895 (3.47)	6955 (4.94)	2940 (2.03)	<0.001
Epilepsy	13085 (4.59)	6220 (4.42)	6865 (4.75)	0.059

Human Immunodeficiency virus	530 (0.19)	380 (0.27)	150 (0.10)	<0.001
Hypertension	264760 (92.78)	130060 (92.34)	134700 (93.21)	<0.001
Hypotension	16315 (5.72)	8450 (6.00)	7865 (5.44)	0.004
Hyperlipidaemia	175395 (61.47)	88545 (62.87)	86850 (60.10)	<0.001
Liver disease	6800 (2.38)	3765 (2.67)	3035 (2.10)	<0.001
Malignancy	12680 (4.44)	6780 (4.81)	5900 (4.08)	<0.001
Malnutrition	26910 (9.43)	11870 (8.43)	15040 (10.41)	<0.001
Smoking cigarettes	40015 (14.02)	25335 (17.99)	14680 (10.16)	<0.001
Non-rheumatic valve disease	28285 (9.91)	12470 (8.85)	15815 (10.94)	<0.001
Obesity	46505 (16.30)	22250 (15.80)	24255 (16.78)	0.002
Pneumonia	30250 (10.60)	16025 (11.38)	14225 (9.84)	<0.001
Previous coronary artery bypass graft	36260 (12.71)	24020 (17.05)	12240 (8.47)	<0.001
Psychiatric disease	58415 (20.47)	23245 (16.50)	35170 (24.34)	<0.001
Renal disease	128395 (44.99)	66880 (47.48)	61515 (42.57)	<0.001
Respiratory failure	42720 (14.97)	20185 (14.33)	22535 (15.59)	<0.001
Rheumatic heart disease	18000 (6.31)	6890 (4.89)	11110 (7.69)	<0.001
Sepsis	8655 (3.03)	4655 (3.31)	4000 (2.77)	<0.001
Thyroid	53945 (18.90)	15750 (11.18)	38195 (26.43)	<0.001
Viral hepatitis	2830 (0.99)	1890 (1.34)	940 (0.65)	<0.001
Thrombolysis	36200 (12.69)	18345 (13.02)	17855 (12.36)	0.015
Endovascular thrombectomy	10900 (3.82)	5185 (3.68)	5715 (3.95)	0.090
Patient Admission year, n, (%)				
2016	66620 (23.4)	31985 (22.7)	34635 (24.0)	<0.001
2017	74285 (26.0)	36560 (26.0)	37725 (26.1)	<0.001
2018	55055 (19.3)	27045 (19.2)	28010 (19.4)	<0.001
2019	89395 (31.3)	45255 (32.1)	44140 (30.5)	<0.001

Table S3. Descriptive statistics of AIS patients with HF stratified by racial/ethnic group. HF—Heart Failure.

	Total	White	Black	Hispanic	Asian or Pacific Islander	Other	P Value
N	285355	191385	61615	18460	5985	7910	
Age, y, median (IQR),	76.00 (65.0-85.0)	79.0 (69.0-87.0)	67.00 (57.00-77.00)	73.00 (62.0-83.0)	76.0 (64.0-85.0)	72. (62.0-82.0)	<0.001
Length of stay, d, median (IQR)	4.0 (3.0-7.0)	4.0 (3.0-7.0)	5.0 (3.0-8.0)	5.00 (3.0-8.0)	50 (3.0-8.0)	5.0 (3.0-9.0)	<0.001
Mortality, n, (%)	18315 (6.4)	13375 (7.0)	2725 (4.4)	1230 (6.7)	495 (8.2)	490 (6.2)	<0.001
Sex, n, (%)							
Female	144510 (50.6)	97845 (51.1)	31410 (51.0)	8470 (45.9)	2945 (49.2)	3840 (48.6)	<0.001
Comorbidities, n, (%)							
Atrial Fibrillation	136720 (47.91)	102995 (53.82)	19315 (31.35)	7820 (42.36)	2985 (49.87)	3605 (45.58)	<0.001
Coronary Heart Disease	153745 (53.88)	107185 (56.00)	29390 (47.70)	9920 (53.74)	3165 (52.88)	4085 (51.64)	<0.001
Deep Vein Thrombosis	5320 (1.86)	3040 (1.59)	1615 (2.62)	390 (2.11)	95 (1.59)	180 (2.28)	<0.001
Infective Endocarditis	1660 (0.58)	1225 (0.64)	320 (0.52)	80 (0.43)	15 (0.25)	20 (0.25)	0.045
Peripheral vascular disease	31745 (11.12)	22925 (11.98)	5590 (9.07)	1915 (10.37)	645 (10.78)	670 (8.47)	<0.001
Previous Valve Surgery	11500 (4.03)	9115 (4.76)	1280 (2.08)	625 (3.39)	165 (2.76)	315 (3.98)	<0.001
Ventricular Tachycardia	11720 (4.11)	7185 (3.75)	3465 (5.62)	575 (3.11)	170 (2.84)	325 (4.11)	<0.001
Venous Thromboembolism	21965 (7.70)	14035 (7.33)	5930 (9.62)	1180 (6.39)	265 (4.43)	555 (7.02)	<0.001
Alcoholism	10090 (3.54)	6300 (3.29)	2735 (4.44)	615 (3.33)	130 (2.17)	310 (3.92)	<0.001
Anemia	62855 (22.03)	38985 (20.37)	15720 (25.51)	4665 (25.27)	1660 (27.74)	1825 (23.07)	<0.001
Use of Anticoagulant(s)	51925 (18.20)	37635 (19.66)	8775 (14.24)	3235 (17.52)	980 (16.37)	1300 (16.43)	<0.001
Use of antiplatelet(s)	87540 (30.68)	59695 (31.19)	18845 (30.59)	5170 (28.01)	1705 (28.49)	2125 (26.86)	<0.001
Arthritis	37435 (13.12)	27190 (14.21)	6955 (11.29)	2000 (10.83)	540 (9.02)	750 (9.48)	<0.001
Bleeding disorder	15155 (5.31)	10245 (5.35)	3035 (4.93)	955 (5.17)	445 (7.44)	475 (6.01)	0.003
Chronic lung disease	75230 (26.36)	54335 (28.39)	14280 (23.18)	3755 (20.34)	1115 (18.63)	1745 (22.06)	<0.001
Dementia	42615 (14.93)	30245 (15.80)	7530 (12.22)	2690 (14.57)	900 (15.04)	1250 (15.80)	<0.001
Diabetes mellitus	133300 (46.71)	82130 (42.91)	33130 (53.77)	10740 (58.18)	3215 (53.72)	4085 (51.64)	<0.001
Drug abuse	9895 (3.47)	4470 (2.34)	4295 (6.97)	630 (3.41)	130 (2.17)	370 (4.68)	<0.001
Epilepsy	13085 (4.59)	7820 (4.09)	3860 (6.26)	865 (4.69)	225 (3.76)	315 (3.98)	<0.001

Human Immunodeficiency virus	530 (0.19)	110 (0.06)	355 (0.58)	40 (0.22)	<11	20 (0.25)	<0.001
Hypertension	264760 (92.78)	175600 (91.75)	58750 (95.35)	17505 (94.83)	5575 (93.15)	7330 (92.67)	<0.001
Hypotension	16315 (5.72)	11150 (5.83)	3385 (5.49)	985 (5.34)	405 (6.77)	390 (4.93)	0.121
Hyperlipidaemia	175395 (61.47)	118910 (62.13)	36605 (59.41)	11380 (61.65)	3890 (65.00)	4610 (58.28)	<0.001
Liver disease	6800 (2.38)	4295 (2.24)	1430 (2.32)	655 (3.55)	195 (3.26)	225 (2.84)	<0.001
Malignancy	12680 (4.44)	9215 (4.81)	2345 (3.81)	625 (3.39)	205 (3.43)	290 (3.67)	<0.001
Malnutrition	26910 (9.43)	18025 (9.42)	5880 (9.54)	1595 (8.64)	660 (11.03)	750 (9.48)	0.194
Smoking cigarettes	40015 (14.02)	24700 (12.91)	11875 (19.27)	1915 (10.37)	495 (8.27)	1030 (13.02)	<0.001
Non-rheumatic valve disease	28285 (9.91)	20870 (10.90)	4545 (7.38)	1625 (8.80)	575 (9.61)	670 (8.47)	<0.001
Obesity	46505 (16.30)	29235 (15.28)	12495 (20.28)	3155 (17.09)	505 (8.44)	1115 (14.10)	<0.001
Pneumonia	30250 (10.60)	20960 (10.95)	5525 (8.97)	2100 (11.38)	670 (11.19)	995 (12.58)	<0.001
Previous coronary artery bypass graft	36260 (12.71)	27885 (14.57)	4280 (6.95)	2410 (13.06)	730 (12.20)	955 (12.07)	<0.001
Psychiatric disease	58415 (20.47)	43275 (22.61)	9995 (16.22)	3135 (16.98)	640 (10.69)	1370 (17.32)	<0.001
Renal disease	128395 (44.99)	81385 (42.52)	31860 (51.71)	8430 (45.67)	3120 (52.13)	3600 (45.51)	<0.001
Respiratory failure	42720 (14.97)	29195 (15.25)	8275 (13.43)	2970 (16.09)	945 (15.79)	1335 (16.88)	<0.001
Rheumatic heart disease	18000 (6.31)	12900 (6.74)	3140 (5.10)	1050 (5.69)	470 (7.85)	440 (5.56)	<0.001
Sepsis	8655 (3.03)	5480 (2.86)	1865 (3.03)	690 (3.74)	270 (4.51)	350 (4.42)	<0.001
Thyroid	53945 (18.90)	42445 (22.18)	6280 (10.19)	2940 (15.93)	975 (16.29)	1305 (16.50)	<0.001
Viral hepatitis	2830 (0.99)	1195 (0.62)	1225 (1.99)	175 (0.95)	115 (1.92)	120 (1.52)	<0.001
Thrombolysis	36200 (12.69)	23960 (12.52)	7515 (12.20)	2740 (14.84)	905 (15.12)	1080 (13.65)	<0.001
Endovascular thrombectomy	10900 (3.82)	7160 (3.74)	2290 (3.72)	765 (4.14)	285 (4.76)	400 (5.06)	0.041
Patient Admission year, n, (%)							
2016	66620 (23.4)	45245 (23.7)	13650 (22.2)	4365 (23.7)	1460 (24.4)	1900 (24.0)	<0.001
2017	74285 (26.0)	49550 (25.9)	15515 (25.2)	5430 (29.4)	1735 (29.0)	2055 (26.0)	<0.001
2018	55055 (19.3)	37380 (19.5)	13330 (21.6)	2325 (12.6)	660 (11.0)	1360 (17.2)	<0.001
2019	89395 (31.3)	59210 (30.9)	19120 (31.0)	6340 (34.3)	2130 (35.6)	2595 (32.8)	<0.001

Table S4. The results of the multivariate analysis showing of the association between HF and receipt of Intravenous Thrombolysis stratified by race/ethnicity in acute ischaemic stroke patients with heart failure. Results are reported as (Predicted Probabilities (95% confidence interval (CI))) and (Absolute risk difference (95% CI)). The no HF category is used as a reference. All models were adjusted for age, sex, pre-existing cardiovascular and non-cardiovascular conditions on admission. Statistically significant results are reported with $p < 0.05$. OR—Odds Ratio, CI—Confidence Interval.

Group		Predicted probability in HF patients(%) (95% CI)	Absolute risk difference (%) (95% CI)	P-Value
Race/Ethnicity	White	12.84 (95% CI:12.43, 13.24)	-0.22 (95% CI: -0.62, 0.18)	0.286
	Black	12.68 (95% CI:11.98, 13.38)	1.86 (95% CI: 1.22, 2.50)	<0.001
	Hispanic	15.40 (95% CI:14.18, 16.62)	2.81 (95% CI: 1.54, 4.09)	<0.001
	Asian/Pacific Islander	15.30 (95% CI:13.17, 17.42)	3.54 (95% CI: 1.33, 5.74)	0.002
	Other	13.66 (95% CI:11.96, 15.37)	1.12 (95% CI: -0.66, 2.90)	0.218

Table S5. The results of the multivariate analysis showing of the association between HF and receipt of Endovascular Thrombectomy stratified by race/ethnicity in acute ischaemic stroke patients with heart failure. Results are reported as (Predicted Probabilities (95% confidence interval (CI))) and (Absolute risk difference (95% CI)). The no HF category is used as a reference. All models were adjusted for age, sex, pre-existing cardiovascular and non-cardiovascular conditions on admission. Statistically significant results are reported with $p < 0.05$. OR—Odds Ratio, CI—Confidence Interval.

Group		Predicted probability in HF patients(%) (95% CI)	Absolute risk difference (%) (95% CI)	P-Value
Race/Ethnicity	White	2.79 (95% CI: 2.59, 2.99)	0.19 (95% CI: 0.02, 0.36)	0.033
	Black	3.10 (95% CI: 2.73, 3.48)	0.77 (95% CI: 0.43, 1.12)	<0.001
	Hispanic	3.21 (95% CI: 2.66, 3.77)	0.76 (95% CI: 0.23, 1.30)	0.005
	Asian/Pacific Islander	3.41 (95% CI: 2.49, 4.34)	0.93 (95% CI: -0.04, 1.90)	0.60
	Other	3.52 (95% CI: 2.75, 4.29)	0.76 (95% CI: -0.07, 1.59)	0.071

Table S6. The results of the multivariate analysis showing of the association between HF and Complications at discharge stratified by race/ethnicity and sex in acute ischaemic stroke patients with heart fail-ure. Results are reported as (Predicted Probabilities (95% confidence interval (CI))) and (Absolute risk difference (95% CI)). The no HF category is used as a reference. All models were adjusted for age, pre-existing cardiovascular and non-cardiovascular conditions on admission. Statistically significant results are reported with $p < 0.05$. OR—Odds Ratio, CI—Confidence Interval.

Group		Predicted probability in HF patients(%) (95% CI)	Absolute risk difference (%) (95% CI)	P-Value
Sex	Male	62.69 (95% CI: 62.08, 63.31)	3.92 (95% CI: 3.33, 4.58)	<0.001
	Female	69.56 (95% CI: 68.92, 70.20)	4.98 (95% CI: 4.35, 5.60)	<0.001
Race/Ethnicity	White	66.12 (95% CI: 65.56, 66.67)	5.84 (95% CI: 5.29, 6.40)	<0.001
	Black	68.12 (95% CI: 67.25, 69.00)	1.47 (95% CI: 0.51, 2.45)	0.003
	Hispanic	62.04 (95% CI: 60.40, 63.68)	1.83 (95% CI: 0.12, 3.53)	0.036
	Asian/Pacific Islander	65.47 (95% CI: 62.64, 68.29)	2.39 (95% CI: -0.53, 0.53)	0.109
	Other	64.37 (95% CI: 61.88, 66.86)	2.30 (95% CI: -0.36, 4.96)	0.090

Figures

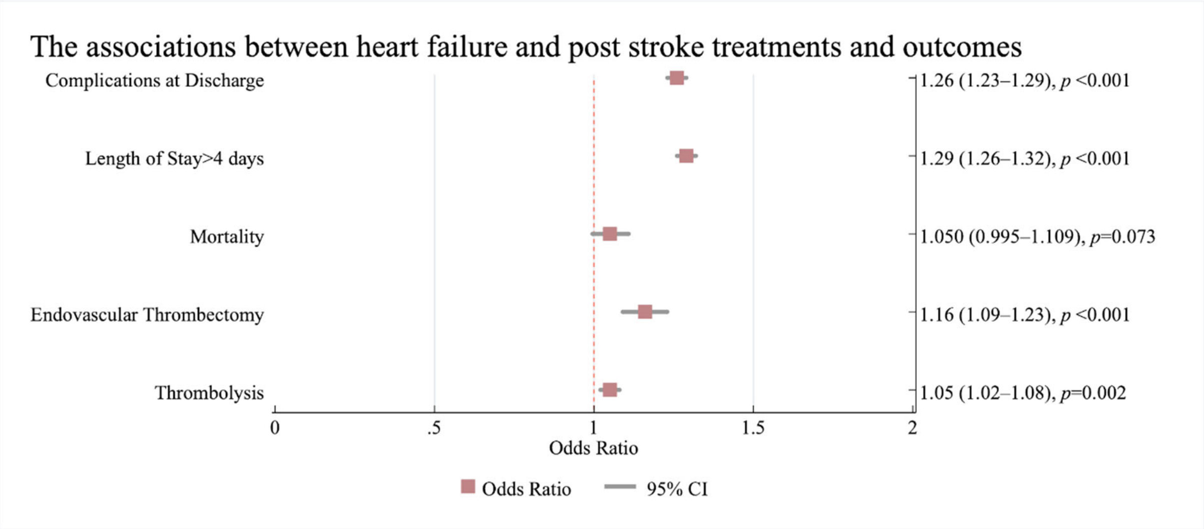

Figure S1. The results of the multivariate logistic regression with no interaction terms. Patients without HF are used as reference. Results are reported as (odds ratio (95% confidence interval). All models were adjusted for age, race/ethnicity, pre-existing cardiovascular and non-cardiovascular conditions on admission. Statistically significant results are reported with $p < 0.05$. Odds ratios and confidence intervals are reported to three decimal places for mortality, where necessary to reflect statistical significance; all other outcomes are reported to two decimal places. AIS—Acute Ischaemic Stroke, LoS—Length of Stay, OR—Odds Ratio, CI—Confidence Interval
