# Supplementary material for: Sex and Ethnic Disparities in Stroke Revascularisation Treatments and Post-Stroke Outcomes in Patients with Heart Failure: A National Inpatient Sample Study
Source: J Clin Med. 2025 Nov 24;14(23):8354. doi: 10.3390/jcm14238354 (PMC12692724; doi:10.3390/jcm14238354)
Supplement: Supplementary file 1 [file jcm-14-08354-s001.zip › Supplement S1.pdf]

SUPPLEMENT S1

Supplement S1.

Table S1. List of the International Classification of Diseases 10th Edition (ICD10) codes utilised to identify covariates

Condition	ICD10 code(s)
Atrial Fibrillation	I480; I481; I4811; I4819; I482; I4820 I4821; I483; I484; I4891; I4892
Coronary Heart Disease	I200; I201; I208; I209; I2101; I2102; I2109; I2111; I2119; I2121; I2129; I213; I214; I219; I21A1; I21A9; I220; I221; I222; I228; I229; I231; I233; I236; I237; I238; I240; I241; I248; I249; I2510; I25110; I25111; I25118; I25119; I252; I253; I2541; I2542; I255; I256; I25700; I25701; I25708; I25709; I25710; I25718; I25719; I25720; I25729; I25758; I25759; I25760; I25799; I25810; I25811; I2582; I2583; I2584; I2589; I259
Deep Vein Thrombosis	I82401; I82402; I82403; I82409; I82411; I82412; I82413; I82419; I82421; I82422; I82423; I82429; I82431; I82432; I82433; I82439; I82441; I82442; I82443; I82449; I82451; I82452; I82453; I82459; I82461; I82462; I82463; I82469; I82491; I82492; I82493; I82499; I824Y1; I824Y2; I824Y3; I824Z1; I824Z2; I824Z3; I824Z9; I82501; I82502; I82503; I82509; I82511; I82512; I82513; I82519; I82521; I82522; I82523; I82531; I82532; I82533; I82539; I82541; I82542; I82543; I82549; I82562; I82591; I82592; I82593; I82599; I825Y1; I825Y2; I825Y9; I825Z1; I825Z2; I825Z3; I825Z9; I82621; I82622; I82623; I82629; I82721; I82722; I82723; I82729; I82A11; I82A12; I82A13; I82A19; I82A21; I82A22; I82A23; I82B11; I82B12; I82B13; I82B19; I82B21; I82B22; I82B29; I82C11; I82C12; I82C13; I82C19; I82C21; I82C22; I82C29
Infective Endocarditis	I330; I339; I38; I39
Peripheral vascular disease	I700; I701; I70201; I70202; I70203; I70208; I70209; I70211; I70212; I70213; I70218; I70219; I70221; I70222; I70223; I70229; I70231; I70232; I70233; I70234; I70235; I70238; I70239; I70242; I70243; I70244; I70245; I70248; I70249; I7025; I70261; I70262; I70263; I70268; I70269; I70291; I70292; I70293; I70298; I70299; I70301; I70302; I70303; I70308; I70311; I70322; I70342; I70362; I70401; I70402; I70413; I70435;

	I70502; I70512; I70621; I70703; I70745; I70749; I708; I7090; I7091; I7092; I7100; I7101; I7102; I7103; I711; I712; I713; I714; I715; I716; I719; I720; I721; I722; I723; I724; I725; I726; I728; I729; I7300; I7301; I731; I7381; I7389; I739; I7409; I7410; I7411; I7419; I742; I743; I744; I745; I748; I749; I75012; I75021; I75022; I75023; I75029; I7589
Previous Valve Surgery	Z952; Z953
Ventricular Tachycardia	I2602; I2609; I2690; I2692; I2693; I2694; I2699; I82401; I82402; I82403; I82409; I82411; I82412; I82413; I82419; I82421; I82422; I82423; I82429; I82431; I82432; I82433; I82439; I82441; I82442; I82443; I82449; I82451; I82452; I82453; I82459; I82461; I82462; I82463; I82469; I82491; I82492; I82493; I82499; I824Y1; I824Y2; I824Y3; I824Z1; I824Z2; I824Z3; I824Z9; I82501; I82502; I82503; I82509; I82511; I82512; I82513; I82519; I82521; I82522; I82523; I82531; I82532; I82533; I82539; I82541; I82542; I82543; I82549; I82562; I82591; I82592; I82593; I82599; I825Y1; I825Y2; I825Y9; I825Z1; I825Z2; I825Z3; I825Z9; I82621; I82622; I82623; I82629; I82721; I82722; I82723; I82729; I82A11; I82A12; I82A13; I82A19; I82A21; I82A22; I82A23; I82B11; I82B12; I82B13; I82B19; I82B21; I82B22; I82B29; I82C11; I82C12; I82C13; I82C19; I82C21; I82C22; I82C29; Z86711; Z86718
Venous Thromboembolism	I2602; I2609; I2690; I2692; I2693; I2694; I2699; I82401; I82402; I82403; I82409; I82411; I82412; I82413; I82419; I82421; I82422; I82423; I82429; I82431; I82432; I82433; I82439; I82441; I82442; I82443; I82449; I82451; I82452; I82453; I82459; I82461; I82462; I82463; I82469; I82491; I82492; I82493; I82499; I824Y1; I824Y2; I824Y3; I824Z1; I824Z2; I824Z3; I824Z9; I82501; I82502; I82503; I82509; I82511; I82512; I82513; I82519; I82521; I82522; I82523; I82531; I82532; I82533; I82539; I82541; I82542; I82543; I82549; I82562; I82591; I82592; I82593; I82599; I825Y1; I825Y2; I825Y9; I825Z1; I825Z2; I825Z3; I825Z9; I82621; I82622; I82623; I82629; I82721; I82722; I82723; I82729; I82A11; I82A12;

	I82A13; I82A19; I82A21; I82A22; I82A23; I82B11; I82B12; I82B13; I82B19; I82B21; I82B22; I82B29; I82C11; I82C12; I82C13; I82C19; I82C21; I82C22; I82C29; Z86711; Z86718
Alcoholism	F1010; F1011; F10120; F10121; F10129; F10151; F10180; F10188; F1019; F1020; F1021; F10220; F10221; F10229; F10230; F10231; F10232; F10239; F1024; F10250; F10251; F10259; F1026; F1027; F10280; F10288; F1029; F10920; F10921; F10929; F1094; F10959; F1096; F1097; F10988; F1099
Anemia	D500; D501; D508; D509; D510; D511; D513; D518; D519; D520; D528; D529; D530; D531; D538; D539; D550; D551; D552; D559; D598; D599; D609; D6101; D611; D612; D61810; D61811; D61818; D619; D62; D630; D631; D638; D642; D643; D644; D6481; D6489; D649
Use of Anticoagulant(s)	Z7901
Use of antiplatelet(s)	Z7902; Z7982
Arthritis	M0000; M00011; M00012; M00032; M00052; M00061; M00062; M00071; M00072; M00231; M00811; M00812; M00821; M00822; M00851; M00861; M00862; M00869; M00871; M00879; M0088; M009; M01X0; M0230; M02371; M02372; M0238; M0239; M041; M042; M048; M0500; M0510; M0519; M0520; M0530; M0540; M0550; M05662; M0570; M05731; M05732; M05741; M05742; M05749; M05761; M05771; M05772; M0579; M0580; M05822; M05829; M05841; M05842; M05851; M05852; M0589; M059; M0600; M06011; M06012; M06031; M06032; M06039; M06041; M06042; M06059; M06071; M06072; M0609; M061; M0630; M064; M0680; M06811; M06812; M06832; M06841; M06842; M06849; M06851; M06859; M06862; M06869; M06871; M0689; M069; M0760; M0800; M0809; M0820; M083; M08451; M0890; M130; M13131; M13132; M13161; M1380; M13811; M13812; M13819; M13821; M13822; M13831; M13832; M13839; M13841; M13842; M13849; M13851; M13852; M13859; M13861; M13862; M13869; M13871; M13872; M13879; M1388; M1389; M150; M151; M152; M153; M154; M158; M159; M160; M1610; M1611; M1612; M162; M1631; M1650; M1651; M167; M169; M170; M1710;

	M1711; M1712; M172; M1730; M1731; M1732; M174; M175; M179; M180; M1810; M1811; M1812; M189; M19011; M19012; M19019; M19021; M19022; M19029; M19031; M19032; M19039; M19041; M19042; M19049; M19071; M19072; M19079; M19111; M19112; M19141; M19142; M19171; M19172; M19179; M19211; M19212; M19219; M19232; M19241; M19242; M1990; M1991; M1992; M1993
Bleeding disorder	D62; D698; D699; G9731; G9732; G9751; G9752; G9761; G9762; H1130; H1131; H1132; H1133; H3560; H3561; H3562; H3563; H4310; H4311; H4312; H4313; H9220; H9221; H9222; H9223; I8501; I8511; I97418; I9742; I97610; I97611; I97618; I9762; I97620; K2211; K226; K250; K254; K256; K260; K262; K264; K266; K274; K276; K280; K284; K2901; K2921; K2931; K2941; K2951; K2961; K2971; K2981; K2991; K31811; K3182; K5521; K625; K9161; K91840; K91841; K920; K921; K922; K9401; K9411; K9421; N938; N939; N950; R040; R041; R042; R0489; R049; R310; R319; R58; S064X9D; S065X0A; S065X0D; S065X0S; S065X1A; S065X1D; S065X2A; S065X4A; S065X6A; S065X9A; S065X9D; S065X9S; S066X0A; S066X0D; S066X0S; S066X1A; S066X1D; S066X2A; S066X3A; S066X8A; S066X9A; S066X9D; S066X9S
Chronic lung disease	J40; J410; J411; J418; J42; J430; J431; J432; J438; J439; J440; J441; J449; J4520; J4521; J4530; J4531; J4540; J4541; J4550; J4551; J45901; J45902; J45909; J45990; J45991; J45998; J470; J471; J479; J60; J61; J628; J632; J634; J64; J662; J668; J670; J672; J679; J680; J684; J688; J689; J8401; J8403; J8410; J84111; J84112; J84113; J84114; J84116; J8417; J8482; J8489; J849
Dementia	F0150; F0151; F0280; F0281; F0390; F0391; G300; G301; G308; G309; G3101; G3109; G311
Diabetes mellitus	E0801; E0810; E0811; E0821; E0822; E0840; E0851; E0859; E08621; E08649; E0865; E089; E0922; E0940; E0942; E0951; E09649; E0965; E099; E1010; E1011; E1021; E1022; E1029; E10311; E10319; E10321; E103212; E103213; E103219; E10329; E103291; E103293; E103299; E103319; E10339; E103399; E103499; E10351; E103511; E103513;

	E103519; E10359; E103592; E103593; E103599; E1036; E1039; E1040; E1041; E1042; E1043; E1044; E1049; E1051; E1052; E1059; E10610; E10620; E10621; E10622; E10628; E10641; E10649; E1065; E1069; E108; E109; E1100; E1101; E1110; E1111; E1121; E1122; E1129; E11311; E11319; E11321; E113211; E113212; E113213; E113219; E11329; E113291; E113292; E113293; E113299; E11331; E113311; E113313; E113319; E11339; E113391; E113392; E113393; E113399; E11341; E113411; E113413; E113419; E11349; E113491; E113492; E113493; E113499; E11351; E113511; E113512; E113513; E113519; E113521; E113522; E113529; E113532; E113542; E113551; E113552; E113553; E113559; E11359; E113591; E113592; E113593; E113599; E1136; E1137X9; E1139; E1140; E1141; E1142; E1143; E1144; E1149; E1151; E1152; E1159; E11610; E11618; E11620; E11621; E11622; E11628; E11630; E11638; E11641; E11649; E1165; E1169; E118; E119; E1300; E1301; E1310; E1311; E1321; E1322; E1329; E13319; E133593; E1339; E1340; E1342; E1343; E1349; E1351; E1352; E1359; E13610; E13621; E13622; E13649; E1365; E1369; E138; E139
Drug abuse	F1110; F1111; F11122; F11129; F1114; F11188; F1119; F1120; F1121; F11220; F11221; F11229; F1123; F1124; F11288; F1129; F1190; F11921; F11929; F1193; F1194; F11988; F1199; F1210; F1211; F12120; F12129; F12159; F12188; F1219; F1220; F1221; F12229; F1223; F12288; F1229; F1290; F12920; F12929; F12959; F12980; F12988; F1299; F1310; F1311; F13120; F13121; F13129; F1314; F1319; F1320; F1321; F13230; F13231; F13232; F13239; F1329; F1390; F13921; F13980; F1399; F1410; F1411; F14120; F14121; F14122; F14129; F1414; F14188; F1419; F1420; F1421; F14222; F14229; F1423; F1424; F14288; F1429; F1490; F14920; F14929; F14959; F14988; F1499; F1510; F1511; F15120; F15121; F15129; F1514; F15150; F15151; F15159; F15182; F15188; F1519; F1520; F1521; F15221; F15229; F1523; F1524; F15280; F15288; F1529; F1590; F15920;

	F15921; F15929; F1593; F1594; F15950; F15959; F15988; F1599; F1610; F1611; F16129; F16188; F1620; F1629; F1690; F1810; F1811; F1890; F18920; F1910; F1911; F19120; F19121; F19129; F1914; F19188; F1919; F1920; F1921; F19230; F19231; F19239; F1929; F1990; F19921; F19929; F19930; F19931; F19939; F1994; F19951; F19959; F19982; F1999
Epilepsy	G40001; G40009; G40011; G40019; G40101; G40109; G40111; G40119; G40201; G40209; G40211; G40219; G40301; G40309; G40311; G40319; G40401; G40409; G40411; G40419; G40501; G40509; G40801; G40802; G40803; G40804; G40811; G40812; G40814; G40822; G40824; G4089; G40901; G40909; G40911; G40919; G40A01; G40A09; G40A11; G40A19; G40B09
Human Immunodeficiency virus	B20
Hypertension	I10; I110; I119; I120; I129; I130; I1310; I1311; I132; I150; I151; I152; I158; I159; I160; I161; I169
Hypotension	I950; I951; I952; I953; I9581; I9589; I959
Hyperlipidaemia	E780; E7800; E7801; E781; E782; E783; E784; E7841; E7849; E785
Liver disease	K700; K7010; K7011; K702; K7030; K7031; K7040; K7041; K709; K710; K7110; K712; K713; K7151; K716; K717; K718; K719; K7200; K7201; K7210; K7211; K7290; K7291; K730; K732; K738; K739; K740; K741; K743; K745; K7460; K7469; K750; K751; K752; K754; K7581; K7589; K759; K760; K761; K763; K766; K767; K7681; K7689; K769; K77
Malignancy	C001; C009; C01; C022; C023; C024; C029; C031; C049; C050; C059; C060; C062; C069; C07; C080; C089; C090; C091; C098; C099; C103; C104; C108; C109; C111; C113; C119; C12; C131; C139; C140; C153; C154; C155; C158; C159; C160; C161; C162; C163; C164; C166; C168; C169; C170; C171; C172; C179; C180; C181; C182; C183; C184; C185; C186; C187; C188; C189; C19; C20; C210; C211; C218; C220; C221; C227; C228; C229; C23; C240; C241; C248; C249; C250; C251; C252; C253; C254; C257; C258; C259; C260; C261; C269; C300; C310; C320; C321; C322; C328; C329; C33; C3400; C3401; C3402; C3410; C3411; C3412; C342; C3430; C3431; C3432;

	C3480; C3481; C3482; C3490; C3491; C3492; C37; C380; C383; C384; C399; C4001; C4020; C4021; C410; C411; C412; C413; C414; C419; C4310; C4321; C4330; C4331; C4339; C434; C4351; C4359; C4360; C4361; C4362; C4371; C4372; C439; C4400; C4401; C4402; C44101; C44109; C44111; C44112; C44119; C441191; C44129; C44202; C44209; C44211; C44212; C44219; C44222; C44229; C44300; C44301; C44309; C44310; C44311; C44319; C44320; C44321; C44329; C4440; C4441; C4442; C4449; C44501; C44509; C44511; C44519; C44520; C44521; C44529; C44599; C44601; C44602; C44609; C44611; C44612; C44619; C44621; C44622; C44629; C44701; C44702; C44709; C44712; C44719; C44722; C44729; C4482; C4490; C4491; C4492; C4499; C450; C451; C457; C459; C460; C467; C469; C479; C480; C481; C482; C490; C4912; C4920; C4921; C4922; C493; C494; C495; C496; C498; C499; C49A0; C49A2; C49A5; C4A30; C4A4; C4A59; C4A61; C4A62; C4A9; C50011; C50012; C50019; C50021; C50022; C50111; C50112; C50119; C50211; C50212; C50311; C50312; C50319; C50411; C50412; C50419; C50511; C50512; C50811; C50812; C50819; C50911; C50912; C50919; C50922; C50929; C518; C519; C52; C530; C531; C538; C539; C541; C542; C548; C549; C55; C561; C562; C569; C5700; C5701; C574; C578; C579; C609; C61; C6290; C6291; C6292; C632; C641; C642; C649; C651; C652; C659; C661; C662; C669; C670; C671; C672; C673; C674; C675; C676; C677; C678; C679; C680; C688; C689; C6900; C6901; C6921; C6930; C6932; C6962; C6981; C6990; C6991; C6992; C700; C709; C710; C711; C712; C713; C714; C715; C716; C717; C718; C719; C720; C7230; C7231; C7259; C729; C73; C7402; C7410; C7412; C7490; C7491; C7492; C751; C753; C755; C760; C761; C762; C763; C7640; C7641; C7650; C7651; C7652; C768; C770; C771; C772; C773; C774; C775; C778; C779; C7800; C7801; C7802; C781; C782; C7839; C784; C785; C786; C787; C7880; C7889; C7900; C7901; C7902; C7911; C7919; C792; C7931; C7932; C7940; C7949; C7951; C7952; C7960; C7961; C7962; C7970; C7971; C7972;

	C7981; C7982; C7989; C799; C7A00; C7A010; C7A019; C7A029; C7A090; C7A092; C7A098; C7A1; C7A8; C7B00; C7B01; C7B02; C7B03; C7B04; C7B09; C7B1; C7B8; C800; C801; C802; C8100; C8101; C8108; C8110; C8119; C8126; C8128; C8140; C8170; C8190; C8191; C8192; C8193; C8194; C8196; C8198; C8199; C8200; C8210; C8213; C8214; C8215; C8219; C8220; C8221; C8222; C8230; C8245; C8280; C8282; C8286; C8288; C8290; C8291; C8293; C8298; C8299; C8300; C8303; C8308; C8310; C8311; C8314; C8318; C8319; C8330; C8331; C8332; C8333; C8334; C8335; C8338; C8339; C8350; C8351; C8358; C8359; C8370; C8371; C8373; C8378; C8379; C8380; C8390; C8400; C8405; C8409; C8410; C8419; C8440; C8444; C8448; C8470; C8490; C84A0; C8510; C8511; C8512; C8513; C8514; C8515; C8516; C8517; C8518; C8519; C8580; C8581; C8582; C8585; C8587; C8589; C8590; C8591; C8592; C8593; C8594; C8595; C8596; C8597; C8598; C8599; C864; C865; C866; C880; C884; C888; C9000; C9001; C9002; C9010; C9030; C9031; C9100; C9101; C9102; C9110; C9111; C9112; C9140; C9141; C9150; C9151; C9160; C9161; C9190; C9191; C9192; C91Z0; C9200; C9201; C9202; C9210; C9211; C9212; C9220; C9240; C9241; C9250; C9290; C9291; C9292; C92Z0; C92Z1; C9300; C9310; C9311; C9400; C9440; C946; C9480; C9481; C9500; C9501; C9510; C9511; C9590; C9591; C966; C969; C96A
Malnutrition	E40; E41; E42; E43; E440; E441; E46; E507; E509; E5111; E5112; E512; E519; E52; E530; E531; E538; E539; E54; E550; E559; E560; E561; E568; E569; E58; E60; E610; E611; E612; E613; E618; E619; E631; E638; E639
Smoking cigarettes	F17200; F17201; F17203; F17208; F17209; F17210; F17211; F17213; F17218; F17219; F17220; F17221; F17223; F17228; F17229; F17290; F17291; F17293; F17299
Non-rheumatic valve disease	I340; I341; I342; I348; I349; I350; I351; I352; I358; I359; I360; I361; I362; I368; I369; I370; I371; I378; I379
Obesity	E6601; E6609; E661; E662; E663; E668; E669
Pneumonia	J13; J14; J150; J151; J1520; J15211; J15212; J1529; J153; J154; J155; J156; J157; J158; J159; J160; J168; J17; J180; J181; J188; J189; J690; J698

Previous coronary artery bypass graft	Z951
Psychiatric disease	F200; F201; F202; F203; F205; F2081; F2089; F209; F21; F22; F23; F24; F250; F251; F258; F259; F28; F29; F302; F308; F309; F310; F3110; F3111; F3112; F3113; F312; F3130; F3131; F3132; F314; F315; F3160; F3161; F3162; F3163; F3164; F3170; F3172; F3173; F3176; F3177; F3181; F3189; F319; F320; F321; F322; F323; F324; F325; F328; F3281; F3289; F329; F330; F331; F332; F333; F3340; F3341; F3342; F338; F339; F340; F341; F3481; F3489; F349; F39; F4000; F4001; F4002; F4010; F4011; F40218; F40231; F40232; F40240; F40241; F408; F409; F410; F411; F413; F418; F419; F42; F423; F424; F428; F429; F430; F4310; F4311; F4312; F4320; F4321; F4322; F4323; F4324; F4325; F4329; F438; F439; F440; F441; F442; F444; F445; F446; F447; F4481; F4489; F449; F450; F451; F4521; F4522; F4541; F4542; F458; F459; F481; F482; F488; F489; F5000; F5001; F5002; F502; F508; F5081; F5089; F509; F5101; F5102; F5104; F5105; F5109; F5111; F5112; F5119; F513; F514; F515; F518; F519; F520; F521; F524; F528; F53; F530; F54; F552; F553; F558; F59; F600; F601; F602; F603; F604; F605; F606; F607; F6081; F6089; F609; F630; F633; F6381; F6389; F639; F640; F641; F648; F649; F659; F6810; F6812; F688; F69
Renal disease	N003; N008; N009; N019; N022; N025; N028; N029; N031; N032; N038; N039; N040; N041; N042; N044; N045; N048; N049; N051; N052; N055; N057; N058; N059; N069; N08; N10; N111; N118; N119; N12; N130; N131; N132; N1330; N1339; N134; N135; N136; N1370; N13729; N138; N139; N140; N141; N142; N143; N144; N151; N158; N159; N16; N170; N171; N172; N178; N179; N181; N182; N183; N184; N185; N186; N189; N19
Respiratory failure	J9600; J9601; J9602; J9610; J9611; J9612; J9620; J9621; J9622; J9690; J9691; J9692
Rheumatic heart disease	I050; I051; I052; I058; I059; I060; I061; I062; I068; I069; I070; I071; I072; I078; I079; I080; I081; I082; I083; I088; I089; I091; I0981; I0989; I099
Sepsis	A400; A401; A403; A408; A409; A4101; A4102; A411; A412; A413; A414; A4150;

	A4151; A4152; A4153; A4159; A4181; A4189; A419
Thyroid	E02; E030; E031; E032; E033; E034; E035; E038; E039; E040; E041; E042; E048; E049; E0500; E0501; E0510; E0520; E0521; E0580; E0581; E0590; E0591; E060; E061; E062; E063; E064; E065; E069; E071; E0781; E0789; E079; E890
Viral hepatitis	B181; B182; B189; B1910; B1920; B1921; B199
Thrombolysis	3E03317; 3E04317; 3E05317; 3E06317; Z9282
Endovascular thrombectomy	03CG3ZZ; 03CG4ZZ; 03CK3Z7

STROBE Statement

	Item No	Recommendation	Page No
**Title and abstract**	1	(*a*) Indicate the study's design with a commonly used term in the title or the abstract	1
		(*b*) Provide in the abstract an informative and balanced summary of what was done and what was found	1
**Introduction**			
Background/rationale	2	Explain the scientific background and rationale for the investigation being reported	1-2
Objectives	3	State specific objectives, including any prespecified hypotheses	2
**Methods**			
Study design	4	Present key elements of study design early in the paper	2
Setting	5	Describe the setting, locations, and relevant dates, including periods of recruitment, exposure, follow-up, and data collection	2
Participants	6	(*a*) *Cohort study*—Give the eligibility criteria, and the sources and methods of selection of participants. Describe methods of follow-up<br>*Case-control study*—Give the eligibility criteria, and the sources and methods of case ascertainment and control selection. Give the rationale for the choice of cases and controls<br>*Cross-sectional study*—Give the eligibility criteria, and the sources and methods of selection of participants	2-3
		(*b*) *Cohort study*—For matched studies, give matching criteria and number of exposed and unexposed<br>*Case-control study*—For matched studies, give matching criteria and the number of controls per case	N/A
Variables	7	Clearly define all outcomes, exposures, predictors, potential confounders, and effect modifiers. Give diagnostic criteria, if applicable	3-5
Data sources/ measurement	8*	For each variable of interest, give sources of data and details of methods of assessment (measurement). Describe comparability of assessment methods if there is more than one group	*2-3*
Bias	9	Describe any efforts to address potential sources of bias	4
Study size	10	Explain how the study size was arrived at	2-3
Quantitative variables	11	Explain how quantitative variables were handled in the analyses. If applicable, describe which groupings were chosen and why	3-5
Statistical methods	12	(*a*) Describe all statistical methods, including those used to control for confounding	7-8
		(*b*) Describe any methods used to examine subgroups and interactions	4-5
		(*c*) Explain how missing data were addressed	2, 4
		(*d*) *Cohort study*—If applicable, explain how loss to follow-up was addressed<br>*Case-control study*—If applicable, explain how matching of cases and controls was addressed<br>*Cross-sectional study*—If applicable, describe analytical methods taking account of sampling strategy	N/A
		(*e*) Describe any sensitivity analyses	4-5

Continued on next page

**Results**			
Participants	13*	(a) Report numbers of individuals at each stage of study—eg numbers potentially eligible, examined for eligibility, confirmed eligible, included in the study, completing follow-up, and analysed	2-3
		(b) Give reasons for non-participation at each stage	N/A
		(c) Consider use of a flow diagram	3
Descriptive data	14*	(a) Give characteristics of study participants (eg demographic, clinical, social) and information on exposures and potential confounders	5-7
		(b) Indicate number of participants with missing data for each variable of interest	2-3
		(c) *Cohort study*—Summarise follow-up time (eg, average and total amount)	N/A
Outcome data	15*	*Cohort study*—Report numbers of outcome events or summary measures over time	*5-16*
		Case-control study—Report numbers in each exposure category, or summary measures of exposure	*N/A*
		Cross-sectional study—Report numbers of outcome events or summary measures	*N/A*
Main results	16	(*a*) Give unadjusted estimates and, if applicable, confounder-adjusted estimates and their precision (eg, 95% confidence interval). Make clear which confounders were adjusted for and why they were included	5-16
		(*b*) Report category boundaries when continuous variables were categorized	2-3, 10
		(*c*) If relevant, consider translating estimates of relative risk into absolute risk for a meaningful time period	10-11
Other analyses	17	Report other analyses done—eg analyses of subgroups and interactions, and sensitivity analyses	10-11
**Discussion**			
Key results	18	Summarise key results with reference to study objectives	11
Limitations	19	Discuss limitations of the study, taking into account sources of potential bias or imprecision. Discuss both direction and magnitude of any potential bias	11-13
Interpretation	20	Give a cautious overall interpretation of results considering objectives, limitations, multiplicity of analyses, results from similar studies, and other relevant evidence	13
Generalisability	21	Discuss the generalisability (external validity) of the study results	11-13
**Other information**			
Funding	22	Give the source of funding and the role of the funders for the present study and, if applicable, for the original study on which the present article is based	14

*Give information separately for cases and controls in case-control studies and, if applicable, for exposed and unexposed groups in cohort and cross-sectional studies.

Note: An Explanation and Elaboration article discusses each checklist item and gives methodological background and published examples of transparent reporting. The STROBE checklist is best used in conjunction with this article (freely available on the Web sites of PLoS Medicine at http://www.plosmedicine.org/, Annals of Internal Medicine at http://www.annals.org/, and Epidemiology at http://www.epidem.com/). Information on the STROBE Initiative is available at www.strobe-statement.org.
